# The Nutritional Pathway Between Tooth Loss and Healthy Ageing: A Longitudinal Study of Older American Adults

**DOI:** 10.3390/nu17040719

**Published:** 2025-02-18

**Authors:** Lujain Sahab, Jonathon Timothy Newton, Wael Sabbah

**Affiliations:** Faculty of Dentistry, Oral and Craniofacial Research, King’s College London, London SE5 8AF, UK; lujain.sahab@kcl.ac.uk (L.S.); tim.newton@kcl.ac.uk (J.T.N.)

**Keywords:** healthy ageing, nutrition, tooth loss, oral health, older adults, longitudinal study, Health and Retirement Study

## Abstract

Objectives: This study examines the mediating role of nutritional intake in the relationship between tooth loss and healthy ageing in older American adults. Methods: A secondary data analysis was conducted using the Health and Retirement Study (HRS), a longitudinal survey of American older adults aged 50 years and over. Data from six waves from 2006 to 2016 were used. Nutritional intake was assessed in 2013 using ten essential nutrients, categorised as adequate or inadequate based on national dietary recommendations. Healthy ageing was an aggregate variable composed of freedom from cognitive impairment, freedom from disability, and high physical functioning. Tooth loss was measured as a dichotomous variable (edentate/dentate). Structural equation modelling was used to assess the associations between tooth loss in 2012, nutrition in 2013, and healthy ageing in 2016, accounting for demographic/socioeconomic factors and behaviours. Results: A total of 3665 participants were included on the analysis. A significant association was found between being dentate in 2012 and nutritional intake in 2013 (coefficient 0.63: 95% CI: 0.62, 0.54, *p* < 0.001). Healthy ageing in 2016 was significantly influenced by socioeconomic factors in 2006 (coefficient 0.84: 95% CI: 1.38, 1.42, *p* < 0.001) and nutrition in 2013 (coefficient 0.05: 95% CI: 0.04, 0.05, *p* < 0.001). Non-Black individuals showed a significant association with healthy ageing. Conclusions: The findings underscore the complex interplay of nutrition, socioeconomic status, health behaviours, and oral health in predicting healthy ageing trajectories. This study highlights the importance of oral health to maintaining adequate nutritional intake, which in turn promotes healthy ageing.

## 1. Introduction

The global population is ageing at an unprecedented rate [1]. Individuals aged 50 and older represent a rapidly growing demographic group, with significant implications for healthcare systems and societal well-being [2].

In the United States, this ageing trend is particularly pronounced, with projections indicating that individuals aged 65 and over will comprise nearly 20% of the population by 2030 [3]. This demographic shift underscores the importance of understanding the factors that contribute to healthy ageing, enabling individuals to live longer and more fulfilling lives [4]. Healthy ageing is a multidimensional concept encompassing physical, cognitive, and social well-being [5]. It is characterised by the ability to maintain functional independence, avoid chronic diseases, and engage in meaningful activities [6]. While genetics play a role, lifestyle factors, particularly nutrition, are increasingly recognised as modifiable determinants of healthy ageing [7].

Nutrition plays a pivotal role in supporting physiological function, maintaining tissue integrity, and mitigating the risk of age-related diseases [8]. Adequate intake of essential nutrients is crucial for preserving muscle mass, bone health, cognitive function, and immune response [9]. Conversely, nutritional deficiencies can accelerate age-related decline and increase vulnerability to chronic conditions such as cardiovascular disease, diabetes, and dementia [10].

Emerging evidence suggests a strong link between oral health and healthy ageing [11]. Oral health, particularly tooth loss, can significantly impact dietary choices and nutritional intake [12]. Tooth loss, a common occurrence in older adults, can lead to difficulties with chewing and mastication, restricting food choices and potentially resulting in inadequate consumption of nutrient-rich foods [13], which in turn could lead to less healthy ageing [7,9]. Studying this pathway between oral health, nutritional intake, and healthy ageing in the same population helps in identifying those at risk of less healthy ageing and enables the development of targeted interventions to support optimal ageing trajectories and promote well-being in the ageing population. However, there is a lack of studies that examined the nutritional pathway between oral health and healthy ageing using longitudinal data. Therefore, this study aims to address this gap by examining the relationships among tooth loss, nutritional intake, and healthy ageing using longitudinal data from the Health and Retirement Study (HRS), a nationally representative sample of older adults in the United States.

## 2. Materials and Methods

### 2.1. Data Source

This study utilised data from the HRS, a nationally representative longitudinal survey of Americans aged 50 and older. Data were extracted from six waves of the biennial HRS survey (2006–2016), and the 2013 Health Care and Nutrition Study (HCNS) module.

### 2.2. Study Sample

The HRS encompasses a vast cohort of more than 43,559 individuals across the United States. This study analysed data from 2006 and included a subset of participants who responded to both the 2013 HCNS module and the 2012 tooth loss question. This resulted in a final analytic sample of 3665 participants.

### 2.3. Study Variables

This study incorporated a range of predictor variables, categorised into demographic factors, behaviours, and socioeconomic factors.

Demographic factors included gender (male/female), age (continuous variable), marital status, which was categorised as married, unmarried/divorced, or widowed and race/ethnicity classified as White American, Black/African American, Hispanic, or other.

Socioeconomic factors comprised education, total wealth, and income. Educational attainment was categorised into four levels: less than high school, high school diploma, some college, and college degree or higher. Total wealth, as defined by the HRS, was reported in nominal dollars and calculated as the sum of assets minus debts. For this analysis, wealth was categorised into quartiles (lowest to highest). The poverty–income ratio, hereafter referred to as “income,” represents the ratio of annual household income to the US Census poverty thresholds. Like wealth, income was also categorised into quartiles.

Two behavioural variables were included: physical activity and smoking status. Physical activity was assessed based on the frequency and intensity of various activities, categorised as light, moderate, or vigorous. Light activities included walking, dancing, and gardening. Moderate activities encompassed tasks like gardening, cleaning the car, and brisk walking. Vigorous activities included aerobics, running, swimming, and cycling. Each activity category was assigned a score based on frequency, with higher scores indicating greater frequency. These scores were then weighted according to intensity (moderate activities weighted ×2, vigorous activities weighted ×3) and summed up to create a composite physical activity scale [14]. Smoking status was categorised as never smoked, former smoker (having smoked at least 100 cigarettes), or current smoker. Body mass index (BMI) was also used in the analysis as a continuous variable as originally reported in the HRS.

Outcome variable: a variable for healthy ageing (HA) was created as an aggregate variable that combined freedom from cognitive impairment, freedom from disability, and high physical functioning [15]. This definition aligns with the World Health Organization’s (WHO) Decade of Healthy Ageing (2021–2030), which emphasizes the importance of maintaining functional abilities and well-being in later life. The WHO framework highlights the multidimensional nature of healthy ageing, encompassing cognitive, physical, and social domains. By incorporating these aspects into the definition of HA, this study adopts a comprehensive approach to assessing healthy ageing that resonates with global efforts to promote well-being in older adults.

Freedom from cognitive impairment was defined as immediate and delayed word recall, serial subtraction, and backwards counting. The ability to perform six activities of daily living (ADL) and five instrumental activities of daily living (IADL) without difficulty was considered freedom from disability. Finally, high physical functioning was defined as the ability to perform 11 daily tasks.

For participants to be considered as free from cognitive impairment they had to have a score ≥12 (out of 27) on a measure involving immediate and delayed word recall, serial subtraction, and backwards counting. Freedom from disability was the ability to perform six activities of daily living (ADL), walking, dressing, bathing or showering, eating, getting in/out of bed, using toilet, and five instrumental activities of daily living (IADL), using the telephone, managing money, taking medication, shopping for groceries, and preparing a hot meal without difficulty. Finally, high physical functioning participants were assessed as having ≤1 difficulty in performing 11 tasks. The tasks included walking one block, walking several blocks, sitting for about 2 h, getting up from a chair after sitting for long periods, climbing one flight of stairs without resting, climbing several flights of stairs without resting, stooping, kneeling, or crouching, reaching or extending arms above shoulder level, pulling or pushing large objects like a living room chair, lifting or carrying weights over 10 pounds, like a heavy bag of groceries, or picking up a dime from a table.

For each of these variables, individuals were categorised as healthy if they were free from cognitive impairment, free from disability, and were high physical functioning. If not, based on the criteria stated before, they were categorised as not healthy. Finally, to create the healthy ageing variable, they were combined and categorised as 0 = healthy if all three variables were healthy, and 1 = not healthy.

### 2.4. Statistical Analysis

To investigate the complex interplay of factors influencing healthy ageing, we utilised structural equation modelling (SEM). This robust statistical approach allowed for the examination of both direct and indirect effects among a network of variables, including latent constructs. SEM was conducted using Mplus Version 8.11 (Muthén and Muthén 1998–2017), with maximum likelihood estimation employed to estimate the model parameters. Model fit was rigorously evaluated using multiple indices, including the chi-square statistic (χ2), comparative fit index (CFI), Tucker–Lewis index (TLI), and root mean square error of approximation (RMSEA). The acceptable thresholds for Mplus indices are greater than or equal to 0.95 for CFI, greater than or equal to 0.90 for TLI, and less than or equal to 0.06 for RMSEA [16].

The model specified a latent variable for socioeconomic factors in 2006, indicated by poverty–income ratio 2006 and wealth 2006. Nutritional intake in 2013 was regressed on tooth loss in 2012. Tooth loss (2012) was, in turn, regressed on smoking status 2012, physical activity 2012, and BMI in 2012. The final model predicted healthy ageing in 2016 based on nutritional intake, smoking status, physical activity, BMI, socioeconomic factors, age in 2006, education, ethnicity, gender, marital status in 2006, and healthy ageing status in 2006 and 2010. The model included standardised estimates, standard errors, and confidence intervals.

## 3. Results

**Figure 1:**
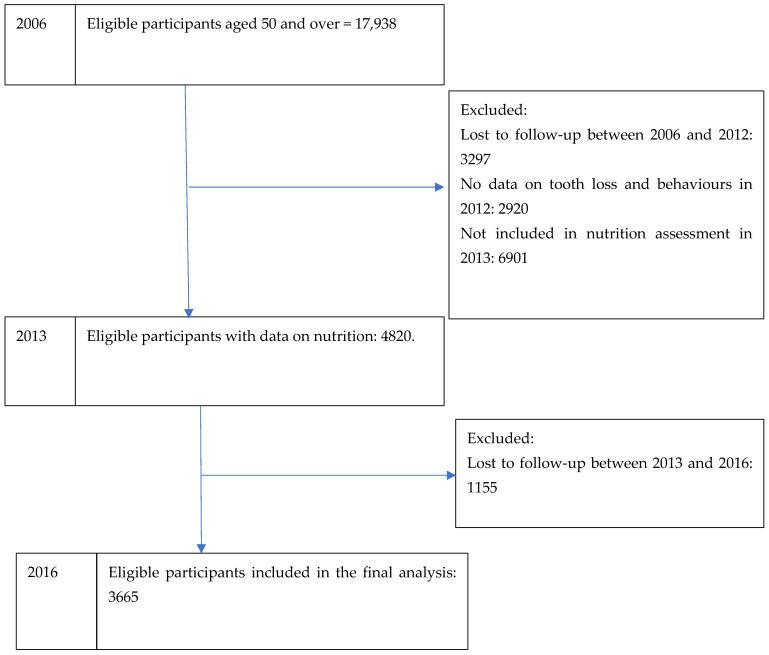
Flowchart of the number of participants from 2006 to 2016.

The analysis included 3665 participants. Figure 1 illustrates a flowchart with the number of participants between 2006 and 2016. The likelihood of individuals aged 50 and over achieving healthy ageing status in 2016 was 40.08%. The percentage of participants with complete tooth loss in 2012 was 16.3%, and the mean total nutrition in 2013 was 3.74 (95% CI: 3.69, 3.78) (Table 1). Further data on the distribution of healthy ageing and other variables can be found in the Supplementary Materials.

**Figure 2:**
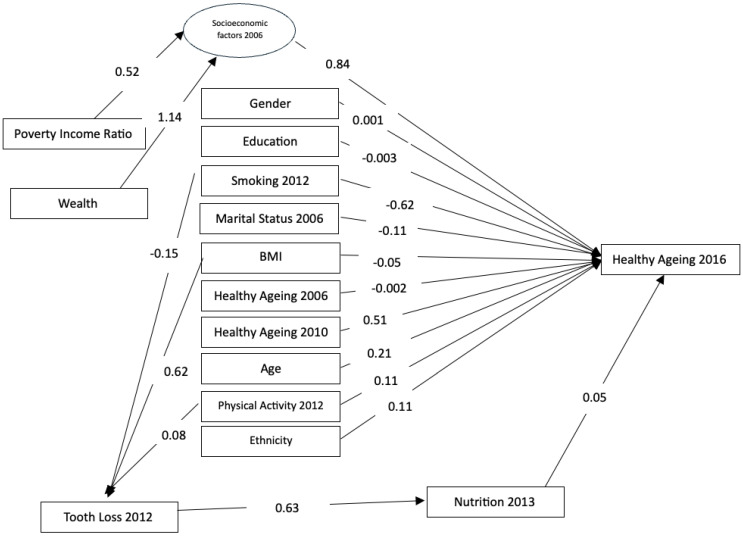
Path diagram presenting the direct and indirect pathways that affect healthy ageing.

Structural equation model (SEM) revealed several key findings. A latent variable representing socioeconomic factors in 2006 was confirmed, with both income (0.52, 95% CI: 1.00, 1.00) and wealth (1.14, 95% CI: 2.13, 2.21) significantly loading onto the construct. A significant association was observed between tooth loss in 2012 and nutritional intake in 2013 (0.63, 95% CI: 0.62, 0.64). Tooth loss (edentulism) was associated with poorer nutritional intake. Smokers in 2012 were more likely to experience tooth loss, since the analysis showed the association with smoking and being dentate (−0.15, 95% CI: −0.15, −0.14), Physical activity (0.08, 95% CI: 0.07, 0.09) was positively associated with being dentate, meaning people who were more physically active were less likely to have tooth loss. A higher BMI was also associated with less likelihood of tooth loss (0.62, 95% CI: 0.58, 0.61) (Figure 2).

In predicting healthy ageing in the final follow-up (2016), socioeconomic factors in 2006 (0.84, 95% CI: 1.38, 1.42) and nutritional intake in 2013 (0.05, 95% CI: 0.04, 0.05) were positively associated with the outcome. This indicates that higher socioeconomic status and healthier nutrition contribute to healthy ageing. Smoking in 2012 was negatively associated with healthy ageing (−0.62, 95% CI: −0.55, −0.54). Physical activity in 2012 was positively associated with healthy ageing (0.11, 95% CI: 0.09, 0.11), while BMI was negatively associated (−0.05, 95% CI: −0.05, −0.04). Age was positively associated with healthy ageing (coefficient: 0.21, 95% CI: 0.18 to 0.20). Education was negatively associated with healthy ageing (−0.003, 95% CI: −0.02, −0.01). Ethnicity was positively associated with healthy ageing (0.11, 95% CI: 0.08, 0.09), indicating that non-White individuals had lower healthy ageing scores. Gender and healthy ageing status in 2006 were not significantly associated with healthy ageing in 2016, but healthy ageing in 2010 was positively associated (0.51, 95% CI: 0.46, 0.47). Marital status in 2006 was negatively associated with healthy ageing (−0.11, 95% CI: −0.10, −0.09). The model fit indices indicated a reasonable fit to the data (RMSEA = 0.55, *p* < 0.001; CFI = 0.33; TLI = 0.05) (Table 2). The significant pathways identified in the model are visually depicted in the path diagram presented in Figure 2.

## 4. Discussion

This study investigated the complex interplay of nutrition, oral health, and socioeconomic and behavioural factors in predicting healthy ageing trajectories among older American adults. Using longitudinal data from the Health and Retirement Study, we found evidence supporting the hypothesised mediating role of nutrition in the relationship between tooth loss and healthy ageing.

Specifically, our analysis revealed that tooth loss had a significant impact on nutritional intake. This finding aligns with previous research demonstrating that oral health, particularly the presence of teeth, can significantly influence dietary choices and nutritional intake [12]. Fukai, Dartevelle [11] argued that tooth loss can lead to difficulties with chewing and food choices, potentially resulting in inadequate consumption of nutrient-rich foods. This is of particular concern since adequate nutrition plays a crucial role in maintaining cognitive function and promoting healthy ageing.

A recent review by Hu [7] highlighted the epidemiological evidence linking various dietary patterns and nutrients to healthy ageing and longevity, with a particular focus on cognitive health. Similarly, Dominguez, Veronese [9] explored the relationship between different dietary patterns and healthy ageing outcomes, including cognitive function, providing evidence supporting the role of healthy diets in preserving cognitive health. The negative impact of tooth loss on nutrition may, therefore, have broader implications for cognitive function and overall well-being in later life.

Furthermore, our model indicated that socioeconomic factors played a crucial role in predicting healthy ageing in later years. This finding echoes the broader literature on social determinants of health, which emphasises the impact of socioeconomic conditions on health outcomes across the lifespan [15].

A systematic review by Wagg, Blyth [17] confirmed a consistent association between higher socioeconomic position and better health outcomes in later life, including reduced risk of chronic diseases, disability, and cognitive decline.

Results showed smoking was negatively associated with healthy ageing and higher likelihood of experiencing tooth loss. This result is consistent with a growing body of evidence demonstrating the detrimental effects of smoking on various aspects of health and well-being in later life [18]. Physical activity was positively associated with healthy ageing. This finding aligns with a substantial body of evidence demonstrating the multifaceted benefits of physical activity for older adults [19].

A key limitation of this study is the timeframe of the data used. While the HRS provides valuable longitudinal information, the final assessment of healthy ageing was in 2016, and nutritional intake was measured in 2013. This limits our ability to account for potential influences of more recent societal shifts, lifestyle changes, and health events that may have impacted the relationships examined. However, our primary aim was to investigate the mediating role of nutritional intake in the link between tooth loss and healthy ageing. These underlying relationships are likely to remain relevant despite evolving lifestyles. Our outcome variable, healthy ageing, was an aggregate measure based on data collected up to 2016. Similarly, nutritional intake was assessed in 2013. These variables, by definition, capture a period preceding the potential influences of more recent events. Also, the study relied primarily on self-reported data from the HRS, which may introduce potential biases.

This study highlights the complex interplay of tooth loss, nutrition, and socioeconomic factors in healthy ageing. Future research should investigate the impact of recent events like the COVID-19 pandemic, explore mechanisms linking tooth loss and nutrition, and examine long-term cognitive effects. Policy recommendations include expanding access to dental care, promoting healthy eating habits, and addressing racial and socioeconomic disparities in healthy ageing. These findings can inform researchers, policymakers, healthcare providers, and public health professionals in developing targeted interventions and policies to support healthy ageing in older adults.

## 5. Conclusions

Overall, this study contributes to a growing body of evidence highlighting the importance of oral health and nutrition in promoting healthy ageing. The findings also underscore the importance of promoting physical activity and smoking cessation interventions as key strategies for enhancing and promoting healthy ageing. The findings also highlight the urgent need to address systematic inequities and promote healthy ageing for all individuals, regardless of race or ethnicity. By addressing these modifiable factors, particularly among socioeconomically disadvantaged populations, we can potentially improve the health and well-being of older adults.

## Figures and Tables

**Table 1:** Characteristics of participants (2006–2016) with distribution of variables (*n* = 3665).

Variables		Mean/Percentage
Healthy ageing 2016 (outcome)		40.08%
Total nutrition (mean) (2013)		3.74 (95% CI: 3.69–3.78)
Age (mean) (2006)		67.85 (95% CI: 67.79–67.92)
Gender	Male	44.27%
	Female	55.73%
Tooth loss (2012)	Dentate	84.06%
	Edentate	15.94%
Wealth (2006)	Highest	25.31%
	Second highest	25.39%
	Second lowest	25.05%
	Lowest	24.24%
Smoking (2012)	Former smoker	42.36%
	Smoker	13.80%

**Table 2:** Path coefficient estimates using SEM.

Variables		Estimate	95% CI	*p*-Value
Socioeconomic factors 2006				
	Poverty income ratio	0.52	(1.00, 1.00)	<0.001
	Wealth	1.14	(2.13, 2.21)	<0.001
Association nutrition 2013				
	Tooth loss (dentate) 2012	0.63	(0.62, 0.64)	<0.001
Association tooth loss (dentate) 2012				
	Smoking 2012	−0.15	(−0.15, −0.14)	<0.001
	Physical activity 2012	0.08	(0.07, 0.09)	<0.001
	BMI 2012	0.62	(0.58, 0.61)	<0.001
Healthy ageing 2016				
	Socioeconomic factors 2006	0.84	(1.38, 1.42)	<0.001
	Nutrition 2013	0.05	(0.04, 0.05)	<0.001
	Smoking 2012	−0.62	(−0.55, −0.54)	<0.001
	Physical activity 12	0.11	(0.09, 0.11)	<0.001
	BMI 2012	−0.05	(−0.05, −0.04)	<0.001
	Age 2006	0.21	(0.18, 0.20)	<0.001
	Education	−0.003	(−0.02, −0.01)	<0.001
	Ethnicity	0.11	(0.08, 0.09)	<0.001
	Gender	0.001	(0.00, 0.02)	NS
	Healthy ageing 2006	−0.002	(−0.002, −0.001)	NS
	Healthy ageing 2010	0.51	(0.46, 0.47)	<0.001
	Marital status 2006	−0.11	(−0.10, −0.09)	<0.001
Model fit				
	RMSEA	0.55		<0.001
	CFI	0.33		
	TLI	0.05		

RMSEA, root mean square error of approximation; CFI, comparative fit index; TLI, Tucker–Lewis index.

## Data Availability

The original contributions presented in this study are included in the article/Supplementary Materials. Further inquiries can be directed to the corresponding author.

## Supplementary Materials

The following supporting information can be downloaded at https://www.mdpi.com/article/10.3390/nu17040719/s1, Distribution of Healthy ageing (HA) and all variables.
