# Supplementary material for: The Nutritional Pathway Between Tooth Loss and Healthy Ageing: A Longitudinal Study of Older American Adults
Source: Nutrients. 2025 Feb 18;17(4):719. doi: 10.3390/nu17040719 (PMC11858001; doi:10.3390/nu17040719)
Supplement: Supplementary file 1 [file nutrients-17-00719-s001.zip › nutrients-3423099-supplementary.pdf]

Supplemental File
Sample Description

Distribution of Healthy ageing (HA) and all variables in Wave 8 (2006) = 17,938 respondents

Variable	Mean (95%CI)	Values	N	Mean within HA (95%CI)	Significance
**HA**	0.38 (0.37-0.39)				
**Age**	68.7 (68.5-68.8)	Not Healthy	8,525	69 (68.8-69.2)	P<0.001
		Healthy	5,244	63.9 (63.7-64.1)	
**BMI**	29 (28.9-29.2)	Not Healthy	3,129	30 (29.8-30.2)	P<0.001
		Healthy	2,311	28.1 (27.9-28.3)	
**Physical Activity**	9.2 (9.1-9.3)	Not Healthy	8,525	8.2 (8-8.3)	P<0.001
		Healthy	5,244	12.4 (12.3-12.6)	

Variable	Values	Mean/Percent (95%CI)	Percentage of HA	Significance
**Gender**	Males	44.3	43.5	p<0.001
	Females	55.8	34.3	
**Ethnicity**	White	65.1	41.4	p<0.001
	Black	18.8	25.7	
	Other	3.8	34.2	
	Hispanic	12.4	30	
**Education**	<Highschool	25.5	16.5	p<0.001
	Highschool/ Equivalent	50.2	37	
	Some college	5.2	47.5	
	College and above	19.2	57.8	
**Smoking**	Current	13.3	33.5	p<0.001
	Former	43.8	37.0	
	Never	42.9	40.6	
**Alcohol**	Excessive	11.3	49.5	p<0.001
	No/Moderate	88.8	36.5	
**Wealth**	Lowest	18.8	16.8	p<0.001
	2nd Lowest	24.4	28.9	
	2nd Highest	28	42.2	
	Highest	28.6	53.5	
**Income**	Lowest	19.7	18	p<0.001
	2nd Lowest	26.6	29.6	
	2nd Highest	27.3	41.8	
	Highest	26.4	56.9	
**Marital Status**	Married	64.5	43.1	p<0.001

	Unmarried/ Divorced *	14.1	35.3	
	Widowed	21.4	22	
**BMI (Cat)****	Obese	38.9	33.4	p<0.001
	Overweight	36.3	48.3	
	Underweight/ Healthy	24.8	48.8	
**Self-Reported Health**	Fair/ Poor	30.4	9.3	p<0.001
	Excellent/ Good	69.7	49	

* Married/spouse absent, Separated, Divorced, Separated/Divorced, never married
** Body Mass Index Using self-reported height and weight, body mass index was categorised as: normal/underweight (< 25; referent), Yes overweight (≥ 25, < 30), obese (≥ 30)

Distribution of Healthy ageing (HA) and all variables in Wave 9 (2008) = 16,861 respondents

Variable	Mean (95%CI)	Values	N	Mean (95%CI)	Significance
**HA**	0.38 (0.37-0.39)				
**Age**	69.7 (69.6-69.9)	Not Healthy	8,078	70 (69.8-70.0)	p<0.001
		Healthy	4,909	65 (64.8 – 65.3)	
**BMI**	29.3 (29.1-29.4)	Not Healthy	2,968	30.3 (30.0 -30.5)	p<0.001
		Healthy	2,005	28.2 (28-28.4)	
**Physical Activity**	9.2 (9-9.3)	Not Healthy	8,078	8.1 (8-8.2)	p<0.001
		Healthy	4,909	12.5 (12.3-12.6)	

Variable	Values	Percent	Percent HA	P-Value
**Gender**	Males	44.2	43	p<0.001
	Females	55.8	34.1	
**Ethnicity**	White	65.2	40.8	p<0.001
	Black	18.7	28.9	
	Other	3.8	36.2	
	Hispanic	12.3	27.3	
**Education**	<Highschool	25.4	15.9	p<0.001
	Highschool/ Equivalent	50.2	36.2	
	Some college	5.2	47.4	
	College and above	19.2	57.9	
**Smoking**	Current	12.6	32	p<0.001
	Former	44.2	36.7	
	Never	43.2	40.8	
**Alcohol**	Excessive	11.4	48	p<0.001
	No/Moderate	88.6	36.4	
**Wealth**	Lowest	19	18.8	p<0.001
	2nd Lowest	24.3	29.4	
	2nd Highest	27.6	39.4	
	Highest	29.2	53.7	
**Income**	Lowest	20	19	p<0.001
	2nd Lowest	26	29.3	
	2nd Highest	27.4	40.8	

	Highest	26.5	56.4	
**Marital Status**	Married	63.4	43	p<0.001
	Unmarried/ Divorced *	14.5	34.2	
	Widowed	22	22.5	
**BMI (Cat)** **	Obese	29.7	31.6	p<0.001
	Overweight	36.1	45	
	Underweight/ Healthy	24.2	48.2	
**Self-Reported Health**	Fair/ Poor	30.7	9.4	p<0.001
	Excellent/ Good	69.3	48.8	

* Married/spouse absent, Separated, Divorced, Separated/Divorced, never married

** Body Mass Index Using self-reported height and weight, body mass index was categorised as: normal/underweight (< 25; referent), Yes overweight (≥ 25, < 30), obese (≥ 30)

Distribution of Healthy ageing (HA) and all variables in Wave 10 (2010) = 21,042

Variable	Mean (95%CI)	Values	N	Mean (95%CI)	Significance
**HA**	0.38 (0.38 – 0.39)				
**Age**	66.6 (66.5-66.8)	Not Healthy	10,967	67.6 (67.4-67.8)	P<0.001
		Healthy	6,818	62 (61.8-62.2)	
**BMI**	29.8 (29.6-29.9)	Not Healthy	4,078	30.6 (30.4-30.8)	P<0.001
		Healthy	2,893	28.8 (28.6-29)	
**Physical Activity**	9.2 (9.1-9.3)	Not Healthy	10,966	7.8 (7.7 – 7.9)	P<0.001
		Healthy	6,818	12.2 (12.1-12.4)	

Variable	Values	Percent	Percent HA	P-Value
**Gender**	Males	44.5	43.1	P<0.001
	Females	55.6	34.8	
**Ethnicity**	White	65.6	41.8	P<0.001
	Black	18.6	29.6	
	Other	3.7	41.5	
	Hispanic	12.1	32.7	
**Education**	<Highschool	25.6	17.3	P<0.001
	Highschool/ Equivalent	50.2	36.2	
	Some college	5.1	46.7	
	College and above	19.1	57.9	
**Smoking**	Current	15.1	31.9	P<0.001
	Former	41.6	37.1	
	Never	43.3	42	
**Alcohol**	Excessive	15	47.9	P<0.001
	No/Moderate	85	36.5	
**Wealth**	Lowest	26.3	24	P<0.001
	2nd Lowest	25.4	33	
	2nd Highest	25.2	43.6	
	Highest	23.1	53.9	
**Income**	Lowest	25	21.7	P<0.001
	2nd Lowest	25.7	30	
	2nd Highest	25.7	43.4	
	Highest	23.6	59.9	

**Marital Status**	Married	63.2	43.4	P<0.001
	Unmarried/ Divorced *	19.2	34.8	
	Widowed	17.7	22.1	
**BMI (Cat)** **	Obese	43.7	34.8	P<0.001
	Overweight	35.3	46.5	
	Underweight/ Healthy	21	47.2	
**Self-Reported Health**	Fair/ Poor	29.9	11.3	P<0.001
	Excellent/ Good	70.1	49.1	

* Married/spouse absent, Separated, Divorced, Separated/Divorced, never married
** Body Mass Index Using self-reported height and weight, body mass index was categorised as: normal/underweight (< 25; referent), Yes overweight (≥ 25, < 30), obese (≥ 30)

Distribution of Healthy ageing (HA) and all variables in Wave 11(2012) = 19,866 respondents

Variable	Mean (95%CI)	Values	N	Mean (95%CI)	Significance
**HA**	0.39 (0.39 – 0.40)				
**Age**	67.6 (67.5-67.8)	Not Healthy	10,215	68.4 (68.2-63.7)	p<0.001
		Healthy	6,614	63.2 (63-63.5)	
**BMI**	29.7 (29.6-29.8)	Not Healthy	3,849	30.6 (30.4-30.8)	p<0.001
		Healthy	2,780	28.7 (28.5-28.9)	
**Physical Activity**	9.3 (9.2-9.4)	Not Healthy	10,214	7.9 (7.8-8)	p<0.001
		Healthy	6,613	12.5 (12.4-12.7)	

Variable	Values	Percent	Percent HA	P-Value
**Gender**	Males	44.3	44.3	p<0.001
	Females	55.7	35.6	
**Ethnicity**	White	65.5	42.3	p<0.001
	Black	18.7	30.3	
	Other	3.7	44.9	
	Hispanic	12.2	33.4	
**Education**	<Highschool	25.5	18	p<0.001
	Highschool/ Equivalent	50.2	36.6	
	Some college	5.1	46.6	
	College and above	19.2	59.6	
**Smoking**	Current	14.2	33.3	p<0.001
	Former	42	37.5	
	Never	43.8	43.3	
**Alcohol**	Excessive	14.3	49.2	p<0.001
	No/Moderate	85.7	37.5	
**Wealth**	Lowest	26.6	24.9	p<0.001
	2nd Lowest	25.9	34.6	
	2nd Highest	24.4	43.6	
	Highest	23.1	55.9	
**Income**	Lowest	25.9	21.2	p<0.001
	2nd Lowest	25.5	33.1	
	2nd Highest	25.4	45.2	
	Highest	23.1	60.7	
	Married	62.7	44.3	p<0.001

**Marital Status**	Unmarried/ Divorced *	19	36.6	
	Widowed	18.3	22.6	
**BMI (Cat)** **	Obese	43.3	34.1	p<0.001
	Overweight	34.4	47.5	
	Underweight/ Healthy	22.3	49.1	
**Self-Reported Health**	Fair/ Poor	29.6	11	p<0.001
	Excellent/ Good	70.4	50.3	

* Married/spouse absent, Separated, Divorced, Separated/Divorced, never married

** Body Mass Index Using self-reported height and weight, body mass index was categorised as: normal/underweight (< 25; referent), Yes overweight (≥ 25, < 30), obese (≥ 30)

Characteristics of Tooth loss and HA in Wave 11 (2012)

**Variable**	**N**	**Values**	**Percent**	**Percent HA**	**Significance**
**Tooth Loss**	**19,838**	**Edentate**	16.3	18.4	p<0.001
		**Dentate**	83.7	43	

Distribution of Healthy ageing (HA) and all variables in Wave 12 (2014) = 18,290 respondents

Variable	Mean (95%CI)	Values	N	Mean (95%CI)	Significance
**HA**	0.37 (0.36 – 0.38)				
**Age**	68.5 (68.3 – 68.7)	Not Healthy	9,899	69.3 (69.1 – 69.6)	p<0.001
		Healthy	5,906	64.3 (64.1 – 64.6)	
**BMI**	29.9 (29.7 – 30.0)	Not Healthy	3,863	30.6 (30.4 – 30.8)	p<0.001
		Healthy	2,563	28.9 (28.7 – 29.1)	
**Physical Activity**	7.94 (7.85 – 8.02)	Not Healthy	9,898	7.9 (7.8 – 8.0)	p<0.001
		Healthy	5,906	12.2 (12.1 – 12.4)	
**Nutrition**	3.74 (3.69 – 3.78)	Not Healthy	3,906	3.7 (3.6 – 3.8)	p<0.001
		Healthy	2,544	3.8 (3.7 – 3.9)	

Variable	Values	Percent	Percent HA	P-Value
**Gender**	Males	44.2	41.9	p<0.001
	Females	55.8	34.1	
**Ethnicity**	White	65.4	42	p<0.001
	Black	18.7	27.7	
	Other	3.7	39.8	
	Hispanic	12.2	29.6	
**Education**	<Highschool	25.4	15.7	p<0.001
	Highschool/ Equivalent	50.2	34.5	
	Some college	5.1	45.8	
	College and above	19.2	57.6	
**Smoking**	Current	12.8	28.5	p<0.001
	Former	43.0	36.2	
	Never	44.2	41.2	
**Alcohol**	Excessive	14.5	48.3	p<0.001
	No/Moderate	85.5	35.4	
**Wealth**	Lowest	25.7	21.3	p<0.001
	2nd Lowest	25.1	32.2	
	2nd Highest	24.5	42.6	
	Highest	24.8	53.4	
**Income**	Lowest	26.1	18.9	p<0.001

	2nd Lowest	24.7	31.0	
	2nd Highest	24.8	42.5	
	Highest	24.4	59.2	
**Marital Status**	Married	61.4	43.4	$p<0.001$
	Unmarried/ Divorced *	19.1	32.3	
	Widowed	19.5	21.7	
**BMI (Cat)** **	Obese	44.4	34.0	$p<0.001$
	Overweight	34.4	44.7	
	Underweight/ Healthy	21.1	44.6	
**Self-Reported Health**	Fair/ Poor	30.7	10.3	$p<0.001$
	Excellent/ Good	69.3	48.7	

* Married/spouse absent, Separated, Divorced, Separated/Divorced, never married
** Body Mass Index Using self-reported height and weight, body mass index was categorised as: normal/underweight (< 25; referent), Yes overweight (≥ 25, < 30), obese (≥ 30)
)

Distribution of Healthy ageing (HA) and all variables in Wave 13 (2016) = 20,148 respondents

Variable	Mean (95%CI)	Values	N	Mean (95%CI)	Significance
**HA**	0.40 (0.39 -0.40)				
**Age**	66.5 (66.4 – 66.7)	Not Healthy	10,536	67.4 (67.1 – 67.6)	p<0.001
		Healthy	7,048	62.4 (62.2 – 62.6)	
**BMI**	30.1 (30.0 – 30.3)	Not Healthy	3,840	30.9 (30.7 – 31.1)	p<0.001
		Healthy	2,744	29.3 (29.0 – 29.5)	
**Physical Activity**	9.4 (9.3 – 9.5)	Not Healthy	10,532	8.0 (7.9 – 8.1)	p<0.001
		Healthy	7,046	12.4 (12.2 – 12.5)	

Variable	Values	Percent	Percent HA	P-Value
**Gender**	Males	44.4	45.3	p<0.001
	Females	55.7	36.2	
**Ethnicity**	White	65.5	44.0	p<0.001
	Black	18.7	33.3	
	Other	3.7	43.8	
	Hispanic	12.1	34.3	
**Education**	<Highschool	25.5	17.3	p<0.001
	Highschool/ Equivalent	50.2	36.4	
	Some college	5.1	48.4	
	College and above	19.1	60.3	
**Smoking**	Current	14.4	30.6	p<0.001
	Former	40.1	38.0	
	Never	45.5	45.2	
**Alcohol**	Excessive	17.3	49.0	p<0.001
	No/Moderate	82.7	38.1	
**Wealth**	Lowest	27.7	24.7	p<0.001
	2nd Lowest	25.0	35.1	
	2nd Highest	23.2	45.1	
	Highest	24.1	56.9	
**Income**	Lowest	27.3	21.3	p<0.001
	2nd Lowest	23.7	32.0	
	2nd Highest	23.5	46.6	
	Highest	25.6	61.9	
**Marital Status**	Married	60.5	46.1	p<0.001
	Unmarried/ Divorced *	22.7	36.0	

	Widowed	16.9	21.3	
**BMI (Cat)** **	Obese	45.9	35.8	p<0.001
	Overweight	34.2	47.1	
	Underweight/ Healthy	19.8	46.3	
**Self-Reported Health**	Fair/ Poor	31.2	12.6	p<0.001
	Excellent/ Good	68.8	51.9	

* Married/spouse absent, Separated, Divorced, Separated/Divorced, never married

** Body Mass Index Using self-reported height and weight, body mass index was categorised as: normal/underweight (< 25; referent), Yes overweight (≥ 25, < 30), obese (≥ 30)
